# Lower Omega-6–Omega-3 Ratio Increased Milk Production and Had Limited Effects on Early Pregnancy Development in Dairy Cattle

**DOI:** 10.3390/ani16030395

**Published:** 2026-01-27

**Authors:** Santiago Andres Paez Hurtado, Leticia P. Sanglard, Andreia Ferreira Machado, M. Sofia Ortega, Ethel Moreno, Simone E. F. Guimarães, James D. Drouillard, Micheal J. Brouk, Victor E. Gomez-Leon

**Affiliations:** 1 Department of Animal Sciences and Industry, Kansas State University, 1424 Claflin Rd, 126 Weber Hall, Manhattan, KS 66506, USA; spaezh@ksu.edu (S.A.P.H.); jdrouill@ksu.edu (J.D.D.); mbrouk@ksu.edu (M.J.B.); 2 Zoetis, 333 Portage Street, Kalamazoo, MI 49007, USA; leticia.sanglard@zoetis.com; 3 Department of Animal Science, Universidade Federal de Viçosa, Viçosa 36570, MG, Brazil; sfacioni@ufv.br; 4 Department of Animal and Dairy Sciences, University of Wisconsin-Madison, Madison, WI 53703, USA; sofia.ortega@wisc.edu (M.S.O.); esmoreno@wisc.edu (E.M.)

**Keywords:** cattle, dairy, reproduction, pregnancy, omega-3, fatty acids

## Abstract

The aim of this study was to evaluate the effect of the linoleic–α-linolenic acid ratio **(LA:ALA)** on cyclicity, oocyte quality, early pregnancy parameters, milk yield, and composition. Holstein cows were randomized to a 6:1-LA:ALA diet (**Low-OMG3:** n = 3 pens; 11 primiparous, 14 multiparous) or a 2:1-LA:ALA diet (**High-OMG3:** n = 3 pens; 10 primiparous, 14 multiparous). Diets were isocaloric and isonitrogenous and fed between 15 and 140 days in milk **(DIM)**. Data were compared using linear mixed models. As expected, omega-3 concentrations in milk and blood increased in the High- compared to Low-OMG3 cows. No effect of diet was observed on cyclicity by 45DIM or oocyte quality at 50DIM. High-OMG3 cows had larger corpus luteum size (11–32 d post-timed artificial insemination **[TAI]**) and greater blood flow (32–60 d post-TAI) than Low-OMG3 cows. However, there was no effect of diet on progesterone, pregnancy-associated glycoproteins, or conceptus size. High-OMG3 cows produced more milk throughout the study, had greater lactose, and tended to have greater protein yield at 50DIM. In conclusion, decreasing the LA:ALA dietary ratio in lactating dairy cows did not provide evidence of effects on cyclicity, oocyte quality, or other early pregnancy-related parameters, but affected corpus luteum size and blood flow, enhanced milk production, and partially increased protein and lactose yields.

## Simple Summary

Dietary fatty acids can influence milk production and reproductive function in dairy cows. Our objective was to evaluate whether reducing the dietary omega-6–omega-3 ratio by increasing omega-3 content would affect milk production and early pregnancy development in lactating dairy cows. Cows fed the omega-3-enriched diet produced more milk and showed changes in corpus luteum size and blood flow, without effects on hormone concentrations, conceptus-related parameters during early pregnancy, postpartum cyclicity, or oocyte quality. Overall, increasing dietary omega-3 had clear benefits for milk production, but its effects on early reproductive outcomes were limited. These results suggest that omega-3 supplementation can be considered a nutritional tool to improve milk yield; however, expectations for improvements in early pregnancy development should be conservative and require further investigation.

## 1. Introduction

Fatty acids **(FAs)** serve as an energy source and play a crucial role in multiple biological processes, including cellular function, metabolic regulation, and hormone responsiveness [1]. FAs constitute a diverse family of compounds characterized by a carboxylic acid group **(COOH)** at one end (alpha) and a methyl group **(CH_3_)** at the opposite end (omega) [2]. FAs are classified based on the number of double bonds in their structure into saturated (no double bonds), monounsaturated (one double bond), and polyunsaturated FAs (**PUFAs**; two or more double bonds) [3]. PUFAs are further classified as omega-6 and omega-3, depending on the location of the first double bond at the sixth or third carbon counting from the omega end, respectively [3,4]. In typical U.S. dairy cattle diets, linoleic acid (**LA**; C18:2 n-6) and α-linolenic acid (**ALA**; C18:3 n-3) are the predominant dietary sources of omega-6 and omega-3 fatty acids and represent the major PUFAs present in plant-based forages and concentrates.

Total mixed rations **(TMRs)** fed to dairy cattle in modern U.S. operations often present an imbalance between omega-6 and omega-3 FAs since several components in the diet are high in omega-6, and just a few contain omega-3 FAs [5]. Since omega-6 and -3 are associated with pro-inflammatory and anti-inflammatory pathways, respectively [6], this imbalance can contribute to an increased production of pro-inflammatory compounds. In contrast, an omega-6 to omega-3 balance in dairy cattle diets is linked to benefits for health and reproductive performance [5]. For instance, increasing dietary ALA content can decrease the absorption of LA, the precursor of prostaglandin F2α **(PGF)**. Indeed, decreasing the omega-6–omega-3 ratio in the diet of dairy cattle can reduce PGF levels. These PGF reductions likely occur because LA and ALA compete for the same metabolic pathways, influencing their incorporation into cell membranes and the relative availability of fatty acid precursors for COX enzymes involved in prostaglandin synthesis.

The PGF reduction observed during decreased omega-6–omega-3 ratios has been associated with higher progesterone concentrations, which are crucial for maintaining pregnancy. Likewise, reduced PGF has also been associated with delayed corpus luteum **(CL)** regression [7,8], which could play a role in decreasing pregnancy losses. In that sense, pregnancy losses in dairy cattle have been linked to both CL regression and embryo failure [9]. In this context, a recent meta-analysis including nearly 20,000 pregnancy records in dairy cows reported pregnancy losses of approximately 27% before day 30, 20% between days 32–60, and 2% between 60 and 90 days post-AI [10]. Earlier work estimated comparable stages of loss at 36%, 8%, and 2%, respectively [11]. Regardless of the percentage, most pregnancy losses in dairy cattle occur before day 30 post-AI, followed by losses between day 32 and 60, and smaller losses from 60 to 90 days post-timed artificial insemination **(TAI)**.

Previous studies have demonstrated that dietary supplementation with omega-3 can increase first-service conception in primiparous and multiparous dairy cows [12], improve early embryo development [13] and elongation [14], and reduce pregnancy loss [15,16]. The present study investigated whether altering the ratio of LA:ALA in the diet could lead to changes in pregnancy development parameters. Additionally, limited information is available regarding the effects of the dietary LA:ALA ratio on in vivo pregnancy development during the first two months of gestation. Therefore, this study aimed to evaluate the effect of the dietary LA:ALA ratio on oocyte quality and pregnancy development during the first 60 days of gestation in high-producing lactating Holstein cows. Additionally, we assessed milk production and composition. Specifically, we hypothesized that decreasing the dietary LA:ALA ratio would enhance (1) postpartum cyclicity and oocyte quality; (2) pregnancy development-related parameters during the first 60 days of gestation in Holstein cows; and (3) milk production and composition.

## 2. Materials and Methods

### 2.1. Ethics Statement

All animal procedures were conducted in accordance with the Guide for the Care and Use of Agricultural Animals in Research and Teaching (United States Department of Agriculture). The experimental protocol was approved by the Institutional Animal Care and Use Committee **(IACUC)** at Kansas State University on 13 August 2021, under protocol no. 4590.

### 2.2. Cows, Location, and Experimental Design

A total of 49 lactating Holstein cows were enrolled in the study. Eligibility required the absence of apparent reproductive tract abnormalities based on ultrasonography and no evidence of health disorders based on an automated ear-tag activity monitoring system (CowManager B.V., Harmelen, Utrecht, The Netherlands). The experiment was carried out at the Kansas State University Dairy Teaching and Research Center from December 2021 through August 2022. Cows were housed in sand-bedded free-stall barns with roofs over the beds. From May until the end of the experiment in August, heat-abatement strategies (i.e., sprinklers over the feed bunk, fans over the beds, and shades over the stall alleys) were implemented. The herd yearly average milk yield was 44.1 kg/cow per day, with milk protein and fat concentrations of 3.0 g/kg and 3.5 g/kg, respectively. Enrolled cows were milked three times daily.

**Figure 1:**
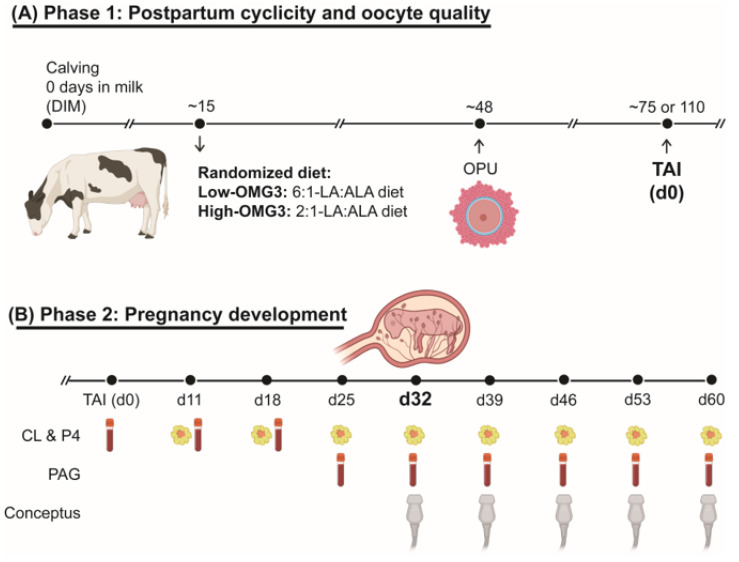
Experimental design of the study: (**A**) Phase 1 comprised the voluntary waiting period: forty-nine lactating dairy cows were enrolled in weekly cohorts at approximately 15 days in milk **(DIM)**. Cows were randomized into receiving a diet with a 6:1-LA:ALA ratio (**Low-OMG3:** n = 3 pens; 11 primiparous, 14 multiparous) or a 2:1-LA:ALA ratio (**High-OMG3:** n = 3 pens; 10 primiparous, 14 multiparous). Diets were isocaloric and isonitrogenous. From 21 to 45 ± 3 DIM, cows were evaluated twice weekly by ultrasound to assess cyclicity resumption post-partum by the presence of the corpus luteum **(CL)**. At 45 ± 3 DIM, all cows received 100 μg of gonadorelin acetate and were submitted to ovum pick-up **(OPU)** at 48 ± 3 DIM for oocyte collection. Subsequently, cows were enrolled at 55 ± 3 DIM into a synchronization protocol for the TAI first service at 75 ± 3 DIM. The protocol consisted of 500 mcg of cloprostenol sodium, followed 3 days later by 100 mcg of gonadorelin acetate, and seven days later by an Ovsynch-56 (with two full doses of cloprostenol). Blood and milk samples were also collected at 15, 50, 75, 110, and 140 DIM to determine the plasma and milk fatty acid profiles and milk components. Milk weights were recorded at each milking. (**B**) Phase 2 comprised the pregnancy development assessments. Briefly, weekly blood samples were collected to assess progesterone **(P4)** and pregnancy-associated glycoprotein **(PAG)** concentrations from 11 days post-TAI until pregnancy diagnosis at 32 days post-TAI. Ultrasound examinations were performed at the same time to measure CL diameter and blood flow. If a cow was diagnosed pregnant at 32 days post-TAI, weekly blood samples were collected until 60 days post-TAI. From 32 to 60 days post-TAI, weekly ultrasound examinations included conceptus and amniotic vesicle size. Additionally, body condition score was evaluated (1 to 5 units) at 21 days before expected calving, calving, 21 days after calving, OPU, TAI, and 30 and 60 days of gestation.

Figure 1 illustrates the study’s experimental design. The study followed a randomized complete block design with parity (primiparous and multiparous) as the blocking factor. Cows were enrolled within the first three weeks after calving and assigned to one of six adjacent pens (three pens per dietary treatment). At any given time, enrollment occurred in two pens (one per diet) until pen capacity was reached, after which enrollment proceeded to the next pair of pens. Because enrollment occurred over approximately 12 weeks and cows remained in the study for ~140 days, all pens were occupied concurrently for part of the experimental period, although cows were at different study days. The two experimental diets were balanced to deliver a 6:1-LA:ALA ratio (**Low-OMG3:** n = 3 pens; 11 primiparous, 14 multiparous) or a 2:1-LA:ALA ratio (**High-OMG3:** n = 3 pens; 10 primiparous, 14 multiparous). The High-OMG3 diet was formulated by incorporating GreatOPlus^®^ into the TMR. GreatOPlus^®^ (Supplementary Materials Table S1) is an extruded feed product produced by Naturally Better Omega-3 (Manhattan, KS, USA) that combines flaxseed, a source of ALA, with *Nannochloropsis oculata*, a source of eicosapentaenoic acid **(EPA)** [17]. Diets were isocaloric and isonitrogenous (Table 1), and the expected intake for the fatty acid composition of the formulated diets is shown in Table 2. Samples of individual dietary ingredients were collected on a weekly basis throughout the experimental period and stored at −20 °C until analysis. Composite samples of all ingredients, prepared from two months of weekly collections during the eight-month study, were analyzed for nutrient composition by near-infrared reflectance spectroscopy at a commercial laboratory (Dairy One, Ithaca, NY, USA). Regardless of experimental diet, TMR was provided once daily and formulated to meet or exceed nutrient requirements for lactating Holstein cows producing 50 kg/d of milk at 3.5% fat and 3.1% true protein, with an anticipated dry matter intake of 24 kg/d [18]. Water and salt mineral blocks were available ad libitum to all cows. Data collection for each cow continued until at least one of the following criteria was met: (1) 60 days of gestation; (2) a non-pregnant diagnosis after the second TAI; or (3) a pregnancy loss diagnosis. Cows remained in the experimental pens even if they were removed from the study. 

### 2.3. Body Condition Score and Postpartum Cyclicity

Body condition was assessed using a 5-point scale (1 = thin; 5 = obese) with 0.25-unit increments. Assessments were performed at calving, at 21 days after calving, on the day of ovum pickup (**OPU**; 48 DIM), at approximately 75 and 110 DIM during first and second services, and at 30 and 60 days of gestation. All cows were evaluated via transrectal ultrasonography twice weekly between 21 and 45 ± 3 DIM to determine resumption of postpartum cyclicity based on the presence of a CL. All ultrasound examinations in the study were performed in a palpation rail using an Ibex-Evo II (E.I. Medical Imaging, Loveland, CO, USA), equipped with a multilinear-array transducer set up at 7.5 MHz.

### 2.4. Blood and Milk Sample Collections

Throughout the experiment, blood and milk samples were collected at 15, 50, 75, 110, and 140 ± 3 DIM. These time points correspond to the enrollment, OPU, and pregnancy check events, respectively. Blood samples were obtained via coccygeal venipuncture using evacuated tubes containing sodium heparin (Vacutainer, Becton Dickinson, and Co., Franklin Lakes, NJ, USA) and immediately placed on ice. Plasma was separated by centrifugation at 1800× *g* using a J-6B centrifuge (Beckman Coulter Inc., Brea, CA, USA) and stored at −20 °C until analysis. Milk samples were collected from the first milking of the day in the milking parlor using plastic sampling bottles attached directly to the milk line. Samples were gently mixed, and subsamples were transferred into two vials: one for milk component analysis and one for fatty acid analysis (stored at −20 °C until assayed).

### 2.5. Milk Yield, Components, and Fatty Acid Profiles in Blood and Milk

Milk yield was recorded at each milking from enrollment until removal from the study. Daily milk weights were converted from pounds to kilograms, and weekly means were calculated for statistical analysis. Milk samples collected at enrollment (used as a covariate) and at 50, 75, 110, and 140 DIM were submitted to the Dairy Herd Improvement Laboratory (MQT Lab Services, Kansas City, MO, USA) for determination of milk composition, i.e., protein [kg], fat [kg], lactose [kg], and somatic cell count.

Plasma obtained from the blood samples at enrollment, 50, 75, 110, and 140 DIM, and milk samples from enrollment and 50 DIM were used to determine fatty acid profiles in blood and milk. Briefly, milk (200 µL) and plasma (500 µL) samples were analyzed for long-chain fatty acid composition with minor modifications to the procedures described by [17] and in the Supelco Technical Bulletin #T496125B [19]. Samples were frozen, freeze-dried overnight, and methylated with 1 mL of benzene containing methyl-C13 internal standard (2 mg/mL) and 4 mL of boron trifluoride–methanol reagent (Supelco, Bellefonte, PA, USA; catalog #B1252). Tubes were gassed with nitrogen, capped, and incubated at 60 °C for 60 min. After cooling, 4 mL of water and 1 mL of hexane were added, vortexed, and centrifuged (1000× *g*, 10 min). The upper organic layer (~1–2 mL) was collected for chromatographic analysis. Fatty acid methyl esters were separated with a capillary column (SP-2560 Supelco, Bellefonte, PA, USA; 100 m × 0.25 mm × 0.20 µm film thickness) with helium as the carrier gas at a flow rate of 1 mL/min. Injector and detector temperatures were maintained at 250 °C. The oven temperature program started at 100 °C (5 min hold), increased at 3 °C per minute to 240 °C, and was held for 20 min.

Individual fatty acid peaks were identified using the Supelco 37-component fatty acid methyl ester reference mixture (catalog #47885-U) and conjugated linoleic acid standards (Matreya, State College, PA, USA; catalog #1255, 1257, 1256, 1254). Response factors for conjugated linoleic acid isomers were derived from the linoleic acid (C18:2) peak average, and for cis-11 octadecenoic acid (C18:1 cis-11) and trans-11 octadecenoic acid (C18:1 trans-11) from cis-9 octadecenoic acid (C18:1 cis-9) and trans-9 octadecenoic acid (C18:1 trans-9), respectively. Docosapentaenoic acid (C22:5 n-3) was identified using a marine polyunsaturated fatty acid reference standard (Sigma, catalog #47033), and its concentration was estimated assuming the same detector response as docosahexaenoic acid (C22:6 n-3).

### 2.6. Oocyte Quality

An intramuscular dose of gonadorelin acetate (100 mcg; 1 mL GONAbreed, Parnell Technologies PTY, LTD, Alexandria, New South Wales, Australia) was administered at 45 ± 3 DIM to synchronize a follicular wave prior to a single **OPU** at 48 ± 3 DIM. Briefly, cows were restrained in a chute, and the area around the first and second caudal vertebrae was clipped and disinfected. Epidural anesthesia was achieved using 3.5 mL of lidocaine administered in the intercoccygeal space. Follicles estimated to measure ≥ 3 mm in diameter were aspirated using an ultrasound-guided transvaginal approach [20] coupled to a vacuum system operating at a continuous negative pressure of 72 mmHg and fitted with a 21-gauge needle. Follicular contents were collected into sterile 50 mL Falcon tubes containing OPU recovery medium (BoviPlus^®^, Minitube, Tiefenbach, Germany) at 38 °C. The recovered fluid was diluted and filtered to facilitate oocyte identification under a stereomicroscope. Recovered oocytes were counted and morphologically evaluated, and cumulus–oocyte complexes were classified according to International Embryo Technology Society guidelines [21] as grade 1 (compact cumulus with >3 cell layers), grade 2 (partial or <3 cumulus cell layers), grade 3 (sparse or absent cumulus cells), or degenerated (oocyte damaged).

Following recovery, oocytes from each cow were placed into cryotubes containing maturation medium and transported overnight in a portable incubator maintained at 38 °C for subsequent assessment of lipid content and mitochondrial abundance. Since handling and transport conditions can influence lipid profiles and mitochondrial activity, oocytes were placed in maturation media to help preserve viability and cellular integrity from collection to staining. These parameters were chosen to test whether omega-3 supplementation, known to alter fatty acid composition and oxidative balance, could influence oocyte energy metabolism and quality. Oocyte recovery data (number of follicles aspirated and oocyte grades) are reported for all 49 cows enrolled in the study. Analyses of oocyte mitochondrial activity and lipid content were performed on oocytes collected from cows enrolled between weeks 4 and 12 of the enrollment period, after staining and imaging protocols were fully established. This subset included cows from both dietary treatments and parities (Low-OMG3: 6 primiparous, 12 multiparous; High-OMG3: 5 primiparous, 11 multiparous) and contributed 166 evaluable oocytes (excluding degenerated oocytes). Cows enrolled during weeks 1–3 were used to establish oocyte recovery, transport, and staining logistics and were therefore not included in the fluorescence analyses. Briefly, each oocyte was sequentially stained for both mitochondria and lipids following previously reported methods [22,23].

Matured oocytes were stained with MitoTracker Deep Red at a 200 nM concentration (Invitrogen, Carlsbad, CA, USA) in Medium-199 supplemented with 1% sodium pyruvate and 0.1% gentamicin for 40 min at 38.5 °C. After staining, oocytes were washed three times in PBS supplemented with 0.1% PVP (PBS-PVP) and fixed in 4% paraformaldehyde for 20 min. Oocytes were then co-stained with 10 µg/mL Nile Red and 1 µg/mL Hoechst 33342 for 15 min to label lipid content and nuclei. The Nile Red working solution was prepared from a stock made by dissolving 1 mg of dye in 1 mL DMSO and diluting in PBS-PVP to 10 µg/mL. Oocytes were mounted on slides containing 10 µL of SlowFade Gold antifade reagent (Life Technologies, Carlsbad, CA, USA), covered with a coverslip, and stored at 4 °C until imaging. Individual oocytes were imaged using a Leica DM5500 epifluorescence microscope (Leica Microsystems, Wetzlar, Germany). Fluorescence quantification was performed using ImageJ v1.46r (National Institutes of Health). Background fluorescence was subtracted, and mean fluorescence intensity was measured within a defined region of interest for each oocyte. Mean intensity values were used to estimate mitochondrial activity (MitoTracker Deep Red) and lipid content (Nile Red). Fluorescence intensity was expressed as arbitrary units **(AUs).**

### 2.7. Timed Artificial Insemination and Pregnancy Development-Related Parameters

To prepare cows for first service at 75 ± 3 DIM, a synchronization protocol [24] was initiated at 55 ± 3 DIM. The protocol consisted of 500 mcg of cloprostenol sodium, followed 3 days later by 100 mcg of gonadorelin acetate. Seven days later, an Ovsynch-56 protocol (with two 500 mcg doses of cloprostenol) was used [25]. Pregnancy status was assessed by transrectal ultrasonography at 32 days after TAI based on the detection of an embryo with a heartbeat. Cows identified as nonpregnant were immediately assigned to one of two resynchronization strategies according to ovarian status. Cows bearing a CL were subjected to a Short-Synch protocol [26] (day 0 [day of pregnancy diagnosis] and day 1, 500 mcg of cloprostenol sodium; day 2 [PM]: 100 mcg of gonadorelin acetate; and TAI 16 to 18 h later). Open cows lacking a CL at pregnancy diagnosis but presenting a follicle ≥ 10 mm in diameter [27] were submitted to an Ovsynch-56. Cows were subjected to a single resynchronization event, and only first- and second-service data were included in the analyses.

Pregnancy-related measurements assessed during the second phase of the study are summarized in Figure 1B. Starting on day 11 post-TAI, blood samples were collected weekly, regardless of service number, to determine progesterone and pregnancy-associated glycoprotein **(PAG)** concentrations until pregnancy diagnosis at day 32 post-TAI. Concurrent ultrasonographic evaluations were performed to assess CL diameter and luteal blood flow using Doppler ultrasonography [28]. Cows confirmed pregnant at day 32 post-TAI, based on detection of a viable conceptus with a heartbeat, continued to undergo weekly blood sampling from day 32 to 60 post-TAI for progesterone and PAG analyses. From day 32 through day 53 post-TAI, weekly ultrasonographic examinations were conducted to evaluate CL diameter and blood flow, as well as conceptus morphometric traits: crown–rump length, conceptus width, and amniotic vesicle length and width [28,29]. At day 60 post-TAI, fetal head length and width were recorded instead of whole-fetus measurements due to imaging limitations at this stage of gestation [30]. Volumes of the CL and embryonic vesicle were calculated using the sphere volume equation [V = 4/3 × π × R^3^, where R = (length/2 + width/2)/2]. At each ultrasonographic evaluation, conceptus viability was verified by assessing the heartbeat. Data collection was discontinued in cows experiencing pregnancy loss, defined as the absence of a viable conceptus.

Progesterone concentrations were quantified using a solid-phase radioimmunoassay with antibody-coated tubes and 125I-labeled progesterone (ImmuChem Coated Tube progesterone 125I RIA Kit, MP Biomedicals, Irvine, CA, USA) following the manufacturer’s instructions. Assay sensitivity was 0.07 ng/mL, the intra-assay coefficient of variation was 7.8%, and the inter-assay coefficient of variation was 8.3%. Pregnancy-associated glycoprotein concentrations were determined by ELISA (BioPRYN Flex; BioTracking LLC, Moscow, ID, USA), with minor modifications to the manufacturer’s protocol. Samples from days 18, 25, 39, 46, 53, and 60 were diluted 1:10, whereas samples from day 32 were diluted 1:20 to fit in the kit standard curve. The intra-assay coefficient of variation was 7.7% and the inter-assay coefficient of variation was 9.8%.

### 2.8. Statistical Analyses

A power analysis was performed, taking into account the effects of diet and using the pen as the experimental unit. The sample size calculation was designed to detect a difference in means of 2 kg of milk (46 vs. 48 kg) at a significance level of 0.05, assuming a pen-level variance of 1 (based on the number of animals per pen to reduce pen-level variance). This resulted in a requirement of three pens per treatment group to achieve a statistical power of 70%. The analysis did not account for DIM, parity, or pregnancy-related parameters. Thus, the reproductive outcomes evaluated (cyclicity, oocyte quality, CL size and blood flow, progesterone and PAG concentrations, and conceptus size) were not powered, and the study should be considered exploratory for those endpoints. This approach was chosen because the study aimed to generate effect size estimates to inform future reproductive research.

For body condition score, blood and milk fatty acid profile, milk production, and milk components (protein, fat, lactose, and somatic cell count [**SCC**]), the response value at the beginning of the experiment (15 DIM) was tested for differences between diets to ensure that the effect of diet did not already exist between the groups before the experiment started. The model included the fixed effects of diet, parity, and the interaction between diet-by-parity, and the random effects of pen within the diet and week of enrollment. The effect of diet was not significant for any of the response variables, and the value at the beginning of the experiment was fitted as a covariate in the model for subsequent analyses. Each response variable was analyzed in a separate model with the value at the first day of experiment as a covariate, and diet (Low-OMG3 and High-OMG3), parity (primiparous vs. multiparous), DIM (as a linear and quadratic covariate), and interactions between diet-by-parity and diet-by-DIM as fixed effects. Random effects were cow, pen within diet, and week of enrollment. Pen was the experimental unit in the experiment and accounted for by the random effect of pen within the diet. Because cows were enrolled in the study as they calved, the week in which each cow was enrolled in the study (study week 1 to 12) was also fitted as a random effect. The quadratic effect of DIM was tested for all response variables and removed from the model when not significant (*p* > 0.05). For milk production, DIM was replaced by weeks in milk (WIM), as milk weights were analyzed as weekly averages. Somatic cell count was log-transformed to approach normality. The pregnancy-related variables were CL volume and blood flow, progesterone and PAG concentrations, conceptus size, and vesicle volume, and the effect of DIM was substituted by the effect of days post-TAI. The quadratic effect of days post-TAI was also tested for all response variables and removed from the model when not significant (*p* > 0.05). For oocyte quality traits, for which there were no repeated records, the fixed effects included diet, parity, and the interaction between diet and parity, and the random effects were pen within the diet and the week of enrollment.

Multiple test comparisons were performed with Tukey’s adjustment when one of the main effects or interactions was significantly different. The normality of the residuals for each model was assessed individually using the Shapiro–Wilk test and visual inspection of the Q-Q plot. Differences were considered statistically significant at *p* ≤ 0.05 and tendencies when 0.05 < *p* ≤ 0.10. Results are shown as adjusted means ± standard error of the mean **(SEM)** or 95% confidence interval (for the log-transformed variable). Statistical analyses were conducted using the GLIMMIX procedure in SAS version 9.4 (SAS Institute Inc., Cary, NC, USA). Supplementary Materials Table S2 shows the number of pens, cows, records, and mean lactation number ±SEM for the major analyses conducted in the study.

## 3. Results

### 3.1. Body Condition Score and Fatty Acid Concentrations in Plasma and Milk

Body condition score did not differ between diets (*p* = 0.15). Across diets, body condition score at −21 DIM (3.0 ± 0.07) and at calving (0 DIM; 3.0 ± 0.07) were greater (*p* < 0.001) than at 21 (2.8 ± 0.07), 50 (2.7 ± 0.07), 75 (2.7 ± 0.07), and 110 DIM (2.8 ± 0.07).

**Figure 2:**
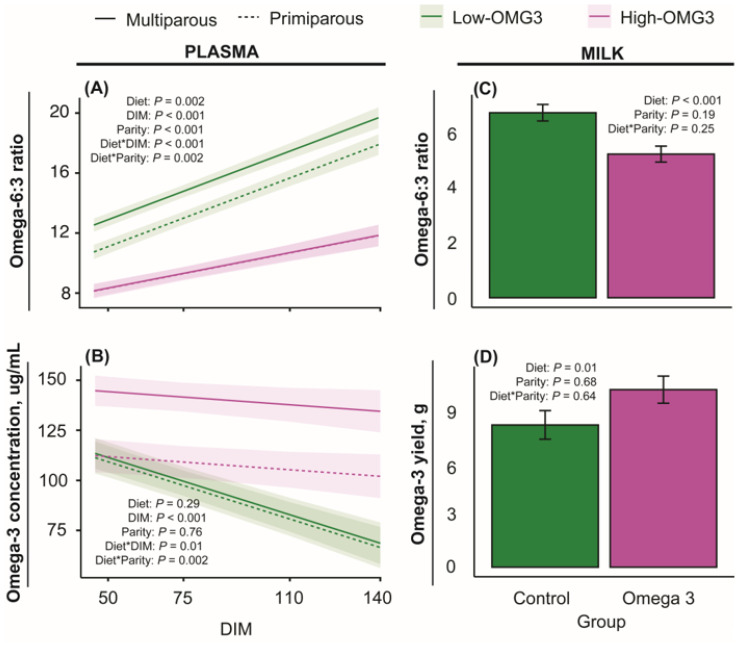
Estimated marginal means ± SEM for omega-6–omega-3 ratio (**top**) and omega-3 concentration and yield (**bottom**) in plasma (**right**) and milk (**left**). Low-OMG3 and High-OMG3 are depicted in green and pink colors, respectively, whereas multiparous and primiparous cows are depicted by a solid or a dashed line, respectively. Data were obtained from Holstein cows receiving a diet with a 6:1-LA:ALA ratio (**Low-OMG3;** green; n = 3 pens, 25 cows) or a 2:1-LA:ALA ratio (**High-OMG3;** pink; n = 3 pens, 24 cows). Diets were isocaloric and isonitrogenous. Statistical analyses were performed within sample type (milk or plasma). Statistical differences (*p* ≤ 0.05) were obtained from Tukey multiple comparison testing within a given DIM and sample type (milk or plasma). An asterisk indicates the interaction between two variables. For the omega-6–omega-3 ratio in plasma (**A**), High-OMG3 was different than Low-OMG3 on days 50, 75, 110, and 140, but the difference increased as DIM progressed. For the omega-3 concentration in plasma (**B**), the Low- and High-OMG3 groups were different from 75 DIM onwards. Moreover, the Low- and High-OMG3 groups differed in the omega-6–omega-3 ratio (**C**) and the omega-3 yield (**D**) in milk samples at 50 DIM.

Figure 2A displays the plasma omega-6–omega-3 ratio obtained from blood samples collected at 50, 75, 110, and 140 DIM. The main effects of diet, DIM, and parity, and the interactions of diet-by-DIM and diet-by-parity, were significant. The diet-by-DIM interaction was primarily explained by a greater ratio in the Low-OMG3 diet compared to the High-OMG3 diet, which increased as DIM increased. The diet-by-parity interaction was primarily due to the greater difference between diets in multiparous cows compared to primiparous cows. Figure 2B displays the omega-3 content in plasma at 50, 75, 110, and 140 DIM. Only the main effect of DIM and the interactions of diet-by-DIM and diet-by-parity were significant. The diet-by-DIM interaction was primarily explained by a greater omega-3 content in the High-OMG3 group and a greater difference between High- and Low-OMG3 as DIM increased. The interaction of diet by parity was explained by multiparous cows in the High-OMG3 diet having a greater omega-3 content than multiparous cows in the Low-OMG3 diet. Figure 2C displays the milk omega-6–omega-3 ratio, whereas Figure 2D displays the milk omega-3 content at 50 DIM. As expected, the ratio of omega-6–omega-3 was significantly lower, and the omega-3 concentration was significantly greater for cows in the High-OMG3 diet than for cows in the Low-OMG3 diet.

### 3.2. Milk Production and Composition

**Figure 3:**
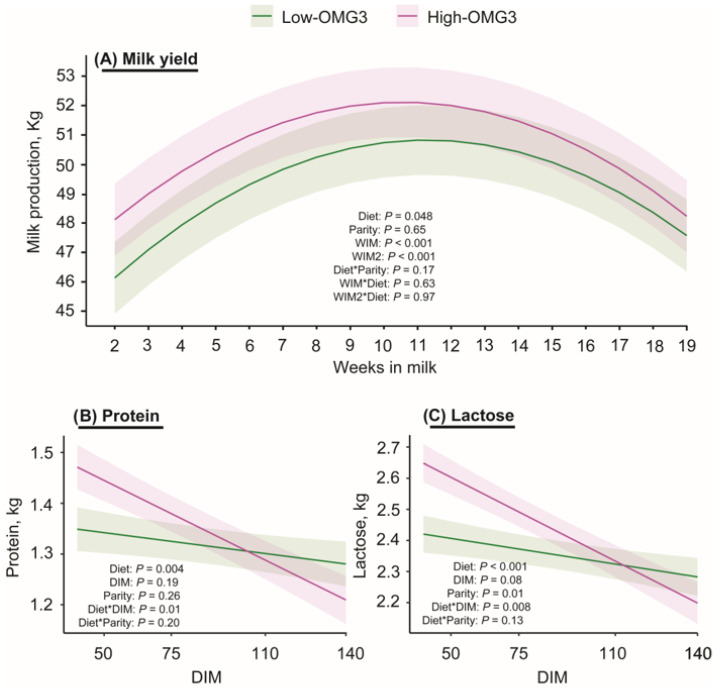
Estimated marginal means ± SEM for (**A**) milk production in kg from cows receiving a diet with a 6:1-LA:ALA ratio (**Low-OMG3;** green; n = 3 pens, 25 cows) or a 2:1-LA:ALA ratio (**High-OMG3;** pink; n = 3 pens, 24 cows). Diets were isocaloric and isonitrogenous. An asterisk indicates the interaction between two variables. The average milk production during the study period was greater in the High-OMG3 cows (50.7 ± 1.2 kg/d) than in Low-OMG3 cows (49.3 ± 1.2 kg/d). (**B**) Depicts least squares means ± SEM for protein yield (kg) for Low- and High-OMG3 cows from samples collected at 50, 75, 110, and 140 DIM. High-OMG3 cows tended to have greater protein yield compared to Low-OMG3 cows only at 50 DIM. (**C**) Depicts least squares means ± SEM for lactose yield (kg) for Low- and High-OMG3 cows from samples collected at 50, 75, 110, and 140. High-OMG3 cows had greater protein yield compared to Low-OMG3 cows only at 50 DIM.

Figure 3 shows the milk production from 2 to 19 weeks in milk **(WIM)**. Milk production averaged 50.7 ± 1.2 and 49.3 ± 1.2 kg/d for High- and Low-OMG3 cows, respectively (*p* = 0.05). As expected, the effects of parity and WIM were also significant, such that multiparous cows had greater milk production, and there was a quadratic effect of WIM. The interactions between diet and WIM, and between diet and parity, were not significant.

Table 3 and Figure 3B,C display the results for milk composition. Significant main effects of diet and diet-by-DIM interactions were observed for protein and lactose yields. The interaction was mainly explained by a tendency to have greater protein yield at 50 DIM and a greater lactose yield at 50 DIM in the High-OMG3 cows than in the Low-OMG3 cows. In contrast, fat yield did not differ between diets. Two cows in the Low-OMG3 and one in the High-OMG3 groups had SCCs between 200,000 and 400,000 cells/mL. Additionally, five cows in the Low-OMG3 and none in the High-OMG3 diet had SCC greater than 400,000 cells/mL. However, SCC did not differ between the Low-OMG3 and High-OMG3 diets. Finally, no main effect of parity or diet × parity interaction was detected (*p* ≥ 0.19) for any of the components. In contrast, the effect of DIM was significantly associated with fat, such that later DIM within the study period was associated with lower fat yield.

### 3.3. Postpartum Cyclicity and Oocyte Quality

Overall postpartum cyclicity, evaluated as the incidence of cows with a CL present between 21 and 45 DIM, was 67% and did not differ between diets. The average day on which the CL was first observed was also not different (*p =* 0.61) between Low-OMG3 cows (26.7 ± 3.1 days) and High-OMG3 cows (28.4 ± 2.9 days). Similarly, the effect of parity was not significant (*p =* 0.83) for the average day on which the CL was first observed postpartum.

**Figure 4:**
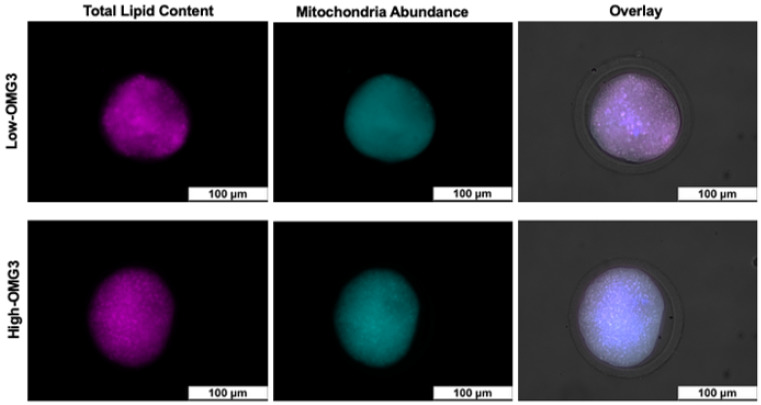
Examples of total lipid content and mitochondria abundance in oocytes from cows receiving a diet with a 6:1-LA:ALA ratio (**Low-OMG3**) or a 2:1-LA:ALA ratio (**High-OMG3**). Diets were isocaloric and isonitrogenous. Mature oocyte lipid content was determined by Nile Red staining, and mitochondria abundance was measured using Mitotracker^TM^ stain. The overlay image is the composite of channels, including brightfield.

Results for the number of aspirated follicles and the quality of recovered oocytes at 48 DIM are summarized in Table 4. There was no effect of parity or diet-by-parity interaction for any of the evaluated parameters: number of aspirated follicles, grade I, II, III, degenerated oocytes, and percentage of recovered oocytes. Although the effect of parity was significant in the initial models for the number of aspirated follicles, grade II oocytes, and degenerated oocytes, multiparous and primiparous cows were not different when tested using the Tukey-adjusted method. Fluorescence intensity analyses were conducted on 166 oocytes as illustrated in Figure 4. The MitoTracker™ Deep Red result, which reflects total mitochondrial abundance, averaged 40.6 ± 8.2 and 42.5 ± 8.7 AU for High-OMG3 and Low-OMG3 cows, respectively, with no difference between diets (*p* ≥ 0.10). The Nile Red result, which reflects intracellular lipid content, averaged 8.0 ± 3.1 and 7.7 ± 3.1 AU for High-OMG3 and Low-OMG3 cows, respectively, and was also not different between diets (*p* ≥ 0.10).

### 3.4. Pregnancy-Development Related Parameters

Progesterone concentrations at day 11 post-TAI did not differ (*p* = 0.71) among all cows submitted to a Low-OMG3 (5.7 ± 0.7) vs. High-OMG3 (5.6 ± 0.8) diet, regardless of pregnancy status.

**Figure 5:**
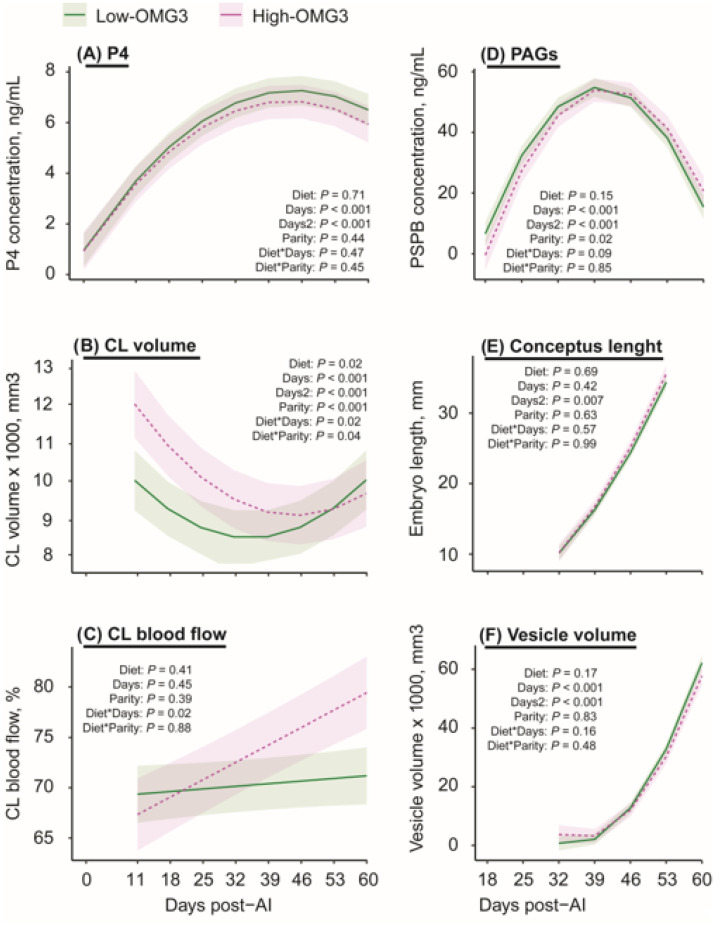
Estimated marginal means ± SEM for pregnancy-related variables from cows receiving a diet with a 6:1-LA:ALA ratio (**Low-OMG3;** green solid line; n = 3 pens, 18 cows) or a 2:1-LA:ALA ratio (**High-OMG3;** pink dashed line; n = 3 pens, 10 cows). Diets were isocaloric and isonitrogenous, fed from 11 to 60 days post-TAI. (**A**) Progesterone **(P4)**, progesterone concentrations in ng/mL; (**B**) Corpus luteum (**CL**) volume in mm^3^; (**C**) CL blood flow in percentage; (**D**) Pregnancy-associated glycoprotein **(PAG)** concentrations in ng/mL; (**E**) Embryo length in mm; and (**F**) Vesicle volume in mm^3^. The only parameter in which diet had an effect was the CL blood flow, in which High-OMG3 cows had greater CL blood flow percentage than the Low-OMG3 cows after day 50 post-TAI. An asterisk indicates the interaction between two variables.

Pregnancy development-related data from the first (54% P/TAI, n = 26/48) and second (36% P/TAI, n = 8/22) inseminations were combined to maximize available observations and compared between the diets. Data from six pregnancy losses were removed from the analysis, and only cows that maintained pregnancy contributed repeated measures through day 60 post-TAI. Pregnancy-related outcomes were evaluated in an exploratory manner. Progesterone concentrations at 0, 11, 18, 25, 32, 39, 46, 53, and 60 days post-TAI (Figure 5A) did not differ (*p* = 0.71) between Low-OMG3 vs. High-OMG3 pregnant cows. There was a quadratic effect (*p* ≤ 0.001) of days post-TAI on progesterone concentrations, which were lower at 0 days post-TAI and increased from days 11 to 60 post-TAI. The interactions of diet-by-day post-TAI for CL volume (*p* = 0.02) and for CL blood flow (*p* = 0.01) in all pregnant cows were significant (Figure 5B,C). A greater CL volume was observed from 11 to 32 days post-TAI in the High-OMG3 than in Low-OMG3 cows, with similar volumes after day 39 post-TAI. In contrast, for CL blood flow, the difference was observed after 32 days post-TAI, when High-OMG3 cows started to show greater blood flow than Low-OMG3 cows. Concentrations of PAG in all pregnant cows (Figure 5D) also did not differ (*p* = 0.14) between pregnant cows submitted to a Low-OMG3 vs. High-OMG3 diet and also showed a significant quadratic effect (*p* ≤ 0.001) of days post-TAI. No effect of diet (*p* ≥ 0.16) was observed on conceptus length (Figure 5E) or vesicle volume (Figure 5F), and both increased over time (*p* ≤ 0.001), as pregnancy progressed. Similarly, fetal head volume on day 60 post-TAI was not different between diets (*p* = 0.27).

## 4. Discussion

Supplementation of dairy cow diets with fat is a common practice in the dairy industry to achieve greater energy density, especially during the transition period [31]. However, commercial fat supplements used in dairy diets typically provide a greater proportion of omega-6 than omega-3 FAs. In addition, other components of the TMR diet are richer in omega-6 than omega-3 [5], intensifying the imbalance and promoting pro-inflammatory pathways [1]. However, previous studies have demonstrated that omega-3 FA can improve early embryo development and decrease PGF secretion, which could extend the CL lifespan. These two mechanisms are of particular interest in our study since pregnancy losses in dairy cattle have been linked to both CL and embryo failure [9]. The present study investigated whether feeding lactating dairy cows a diet enriched in ALA as a means to increase omega-3 could lead to alterations in pregnancy development parameters. The results of the current study demonstrated that decreasing the LA:ALA ratio in the diet of lactating cows enhanced milk yield, protein, and lactose at 50 DIM, CL volume (11 to 32 days post-TAI), and CL blood flow (32 to 60 days post-TAI). However, these changes were not associated with progesterone concentrations, oocyte quality, or the pregnancy-development-related parameters evaluated in this study.

Consistent with the treatment diets, the plasma omega-6–omega-3 ratio was similar between groups at enrollment and decreased in High-OMG3 cows at 50, 75, 110, and 140 DIM. The greater omega-3 concentration obtained from plasma (Figure 2) demonstrates that the High-OMG3 diet, which included flaxseed and algae, can provide absorbable omega-3 content that is available in circulation. Accordingly, High-OMG3 cows exhibited a lower omega-6–omega-3 ratio and greater omega-3 content as early as 50 DIM. This is consistent with previous reports showing that omega-3 fatty acids can be metabolized and deposited in milk within approximately four weeks of dietary intake [13]. A 67% reduction in the omega-6–omega-3 ratio between 15 and 50 DIM was observed in High-OMG3 cows, consistent with the 41% reduction previously reported for rumen-protected, encapsulated fish oil supplementation [32].

Dietary omega-3 supplementation has been associated with increased omega-3 content across multiple tissues in dairy cattle. Reductions in the omega-6–omega-3 ratio have been reported in the endometrium, liver, mammary gland, and muscle following supplementation with calcium salts of fish oil compared with whole cottonseed between 17 and 194 DIM [33,34]. In caruncles, fish oil supplementation has also been shown to increase omega-3 fatty acid content when compared to omega-6-rich lipid sources such as olive oil [8]. In this context, the use of a product such as GreatOPlus^®^, which combines flaxseed and *Nannochloropsis oculata*, provides complementary omega-3 sources: flaxseed supplies ALA, and *Nannochloropsis oculata* serves as a direct source of EPA. This distinction is biologically relevant, given the limited efficiency of endogenous ALA conversion to EPA [35].

Given the established roles of omega-3 and omega-6 fatty acids in modulating anti-inflammatory and pro-inflammatory pathways, we hypothesized that dietary treatment would influence postpartum resumption of cyclicity and oocyte quality. However, our results failed to support the hypothesis regarding the resumption of cyclicity. This outcome aligns with previous reports indicating no differences in the proportion of cyclic cows at 63 DIM following palm oil or fish oil supplementation from 30 to 160 DIM [16], as well as the absence of differences in the timing of first postpartum ovulation in cows supplemented with protected fish oil compared with toasted soybean [32]. In contrast, Sinedino et al. (2017) [12] increased estrus expression in primiparous cows fed an algae-supplemented diet relative to controls, whereas no such effect was observed in multiparous cows, highlighting potential parity-dependent responses to omega-3 supplementation. Our results also failed to support the hypothesis of oocyte quality, as no effect of diet or a diet-by-parity interaction was observed on the evaluated parameters. Within different ovarian compartments (e.g., follicular fluid, granulosa cells, cumulus-oocyte), omega-3 content has been reported to be altered [36], suggesting a specific role or distinct metabolism of PUFAs in these tissues [5]. This selective uptake has been associated with alterations in oocyte quality, as reported in ewes [36] and dairy cow embryos [13,37]. However, our results for oocyte mitochondrial abundance and lipid content did not differ between diets and also did not support the hypothesis. One may consider that we aspirated and synchronized small, growing follicles that may have only been in contact with the follicular fluid for a short period.

The second part of our working hypothesis was primarily not supported. That is, the dietary LA:ALA ratio did not enhance in vivo pregnancy development during the first 60 days of gestation in dairy cows. Evidence partially supporting the hypothesis was observed in the larger CL volume observed between 11 and 32 days post-TAI and greater CL blood flow between 32 and 60 days post-TAI in High-OMG3 compared with Low-OMG3 cows. The larger CL observed in the High-OMG3 group in our study could be attributed to a larger preovulatory follicle [38] during the synchronization protocol; however, we did not evaluate this parameter in our experiment. Previous studies have shown that cows supplemented with omega-3 have greater preovulatory follicle size [15,32], whereas others have not found differences [12,39]. Thus, in our study, it remains unclear whether omega-3 supplementation increased the preovulatory follicle size and, consequently, the CL size.

Despite differences in CL size observed in our study, progesterone concentrations did not differ between diets. The relationship between CL size and progesterone concentration is well established; however, in certain physiological contexts—such as when comparing non-lactating heifers and lactating cows—non-lactating heifers typically have smaller CL but greater progesterone concentrations [40,41]. In lactating dairy cows, elevated milk production is associated with increased feed intake and hepatic blood flow, which in turn enhances the metabolic clearance of steroid hormones such as progesterone [42]. Consequently, circulating progesterone concentrations reflect not only corpus luteum size and function, but also the rate of systemic progesterone metabolism. Under conditions of high hepatic clearance, increases in CL size and luteal blood flow may therefore not translate into higher circulating progesterone concentrations [40,41,42]. This mechanism provides a plausible explanation for the dissociation observed in the present study between greater CL size and blood flow and unchanged progesterone concentrations in High-OMG3 cows, which also exhibited greater milk yield than cows receiving the Low-OMG3 diet.

Reports on the effects of omega-3 supplementation on progesterone concentrations are contrasting in the literature. Several studies have reported no differences in progesterone concentrations when comparing omega-3- and omega-6-enriched diets in lactating dairy cows [12,13,15]. However, others have documented increased progesterone concentrations in response to omega-3 supplementation [43]. In contrast, Hutchinson et al. (2012) [44] reported reduced CL volume and progesterone concentrations in cows fed omega-3 diets. The differences between the studies could be related to the period of supplementation, the timing of evaluating progesterone concentrations, and whether they were evaluated in pregnant and non-pregnant cows or only in one of these categories. We analyzed data from pregnant cows through day 60 of gestation, whereas other studies collected samples on non-bred cows or cows regardless of their pregnancy status [43], or collected samples at days 0, 21, and 24 post-TAI as part of early pregnancy status assessment [15].

We did not observe a difference in PAG concentration between diets. Maternal PAG concentrations in blood have been used as a marker of embryo presence [9]. However, its function remains to be elucidated. At approximately 20 days of gestation in cows, binucleate giant cells originating from the trophoblast migrate to the endometrial epithelium [45] and synthesize PAG until it is released into maternal circulation [46]. Thus, PAG concentrations from 20 to 28 days post-TAI have been associated with pregnancy viability [47]. Although the High-OMG3 diet resulted in a larger CL and greater blood flow during specific times within the first 60 days of gestation, the results did not provide evidence of a direct effect of omega-3 supplementation on conceptus or amniotic vesicle size during this period.

Finally, the last part of our working hypothesis was supported, since decreasing the dietary LA:ALA ratio in lactating cows increased the average milk yield during the study period. Reports on the effects of omega-3 supplementation on milk production are inconsistent in the literature. Some studies have reported no differences between omega-3 diets and control diets [13,15,32,48], while others have observed increased milk production [12,49,50]. It should be noted that in some of those studies, omega-3 and control diets were not isocaloric, unlike in the present study. Contrary to our hypothesis, SCC and fat yield did not differ between diets. Additionally, differences in protein and lactose yields were only observed at 50 DIM but not at later time points. Similarly, no differences in milk protein or fat percentage were reported by Thangavelu et al. (2007) [13] among cows supplemented with flaxseed (rich in omega-3 FA), sunflower (rich in omega-6 FA), or saturated fatty acids, with comparable findings reported for flaxseed versus sunflower supplementation by Ambrose et al. (2006) [15]. A speculative explanation for the increased milk production in omega-3-supplemented cows may be related to reduced energy and nutrient utilization for pro-inflammatory pathways, as suggested by previous studies [6,51,52].

Although dietary omega-3 supplementation increased milk production in the current study, several limitations should be considered when interpreting the reproductive outcomes reported herein. The experimental design and power analysis were based on milk yield, using pen as the experimental unit, and the study was not powered to detect small differences in reproductive endpoints; therefore, conclusions regarding cyclicity, corpus luteum characteristics, oocyte quality, and early pregnancy development should be interpreted cautiously. These results should be viewed as effect-size estimates to inform future reproductive research. Pregnancy data from first and second timed artificial inseminations were pooled to maximize available observations, which may have masked biologically relevant differences associated with service number or stage of lactation. In addition, oocyte quality was assessed following synchronization and aspiration of relatively small follicles at approximately 48 DIM, a timing that may limit the ability to detect dietary effects on oocyte competence. Mitochondrial activity and lipid content were evaluated in a subset of cows after protocol standardization, and oocytes were transported overnight under controlled conditions, which may influence absolute fluorescence measurements. Furthermore, the observed increases in corpus luteum size and blood flow without corresponding changes in circulating progesterone suggest that luteal structural or vascular changes do not necessarily translate into measurable endocrine differences, potentially due to factors such as progesterone metabolic clearance. However, the modest number of pens per treatment (n = 3) inherently limits pen-level inference and generalizability, despite appropriate statistical modeling. Collectively, these considerations support a conservative interpretation of the limited effects of omega-3 on early pregnancy development and reproductive outcomes, while reinforcing the robustness of the observed milk production response and underscoring the need for additional research before applying LA:ALA ratio modifications as a reproductive strategy in dairy herds.

## 5. Conclusions

As expected, the omega-6–omega-3 ratio was reduced in the milk and plasma samples of cows on a reduced dietary LA:ALA ratio when compared with cows fed a greater LA:ALA ratio diet. The reduced LA:ALA diet was achieved mainly by increasing dietary ALA content, one of the primary omega-3 fatty acids. Thus, omega-3 concentration and yield were also greater in the plasma and milk of High-OMG3 cows, indicating that dietary omega-3 fatty acids were absorbed and distributed across multiple body tissues. Overall, dairy cows fed the omega-3 FA-enriched diet averaged greater milk production during the study period when compared with cows on a low-omega-3 diet. High-OMG3 cows also had greater protein and lactose yields at 50 DIM, without differences in fat yield or somatic cell count. However, diet did not affect resumption of postpartum cyclicity or oocyte quality. Among pregnancy development-related parameters, the corpus luteum size between 11 and 32 days post-TAI and CL blood flow between 32 and 60 days post-TAI were also increased in the high-omega-3 cows. However, none of the other reproductive parameters differed between diets. Altogether, the benefits of feeding omega-3 for pregnancy development parameters during the first 60 days of gestation in lactating Holsteins were related only to the corpus luteum, but this did not translate into greater progesterone concentrations, pregnancy-associated glycoprotein concentrations, or conceptus and embryonic vesicle sizes. Although the dietary intervention showed promising effects on milk yield, limited power for reproductive endpoints underscores the need for additional research before applying LA:ALA ratio modifications as a reproductive strategy in dairy herds.

## Figures and Tables

**Table 1:** Dietary ingredients and nutrient composition of the diets used in the study.

Item		Diets	
		Low-OMG3	High-OMG3
Ingredients, % of dry matter			
	Corn silage	34.7	32.8
	Alfalfa hay	19	19
	Corn grain ground	15.3	15.9
	Wet corn gluten feed	17.7	17.7
	Whole cottonseed	1	0
	Soy Plus	8.5	7.7
	GreatOPLUS	0	3.5
	Megalac	0.6	0
	Sodium bicarbonate	1.1	1.1
	MKC dairy micro	1.3	1.3
	Calcium carbonate	1	1
Nutrients			
	Net energy for lactation, Mcal/kg	1.7	1.7
	Crude protein, %	17.5	17.5
	Starch, %	22.9	23.5
	Nonfibrous carbohydrate, %	36.9	38.1
	Acid detergent fiber, %	18.3	18.1
	Neutral detergent fiber(NDF), %	31.4	30.3
	NDF from forage, %	18.4	17.7
	Ether extract, %	4.7	4.3
	Ca, %	1.1	1.1
	P, %	0.5	0.5
	Mg, %	0.4	0.4
	K, %	1.6	1.6
	Na, %	0.6	0.7
	Omega-6–omega-3 ratio	6:1	2:1

**Table 2:** Expected intake on g/day per cow for the fatty acid composition of a diet formulated to deliver a 6:1-LA:ALA ratio (Low-OMG3) or a 2:1-LA:ALA ratio (High-OMG3).

Fatty Acid (Common Name)	Low-OMG3	High-OMG3	Fatty Acid Family
Lauric acid (C12:0)	2.11	1.78	Saturated
Myristic acid (C14:0)	8.07	5.47	Saturated
Palmitic acid (C16:0)	193.14	113.92	Saturated
Palmitoleic acid (C16:1)	3.05	7.54	Monounsaturated
Stearic acid (C18:0)	27.42	27.23	Saturated
Elaidic acid (C18:1 trans)	0.35	0.34	Monounsaturated (trans)
Oleic acid (C18:1 cis)	178.91	150.27	Monounsaturated (cis)
Other fatty acids	17.35	16.05	Mixed
Linoleic acid (C18:2) ^A^	341.04	321.48	Polyunsaturated (n-6)
α-Linolenic acid (C18:3) ^B^	56.68	158.33	Polyunsaturated (n-3)
LA:ALA ratio	6.0	2.0	-

LA:ALA ratio calculated as A/B (C18:2/C18:3); Diets were isocaloric and isonitrogenous.

**Table 3:** Least squares mean (±SEM) for milk composition from Holstein cows receiving a diet with a 6:1-LA:ALA ratio (Low-OMG3; n = 3 pens, 25 cows) or a 2:1-LA:ALA ratio (High-OMG3; n = 3 pens, 24 cows).

Endpoint	Diet ^1^		*p*-Value				
	Low-OMG3	High-OMG3	Diet	Parity	DIM	Diet x Parity	Diet × DIM
Fat, kg	1.43 (0.05)	1.41 (0.05)	0.14	0.11	0.02	0.66	0.08
Protein, kg	1.32 (0.03)	1.35 (0.03)	0.004	0.25	0.19	0.20	0.01
SCC ^2^	31.5 [11.7; 82.0]	20.8 [7.9; 52.0]	0.74	0.52	0.39	0.45	0.20
Lactose, kg	2.37 (0.05)	2.44 (0.05)	0.001	<0.001	0.17	0.19	0.008

^1^ Cows received a diet with a 6:1-LA:ALA ratio (**Low-OMG3**) or a 2:1-LA:ALA ratio (**High-OMG3**). Diets were isocaloric and isonitrogenous; ^2^ For somatic cell count (SCC), the SEM was substitute by 95% confidence interval and the values were back-transformed from log scale.

**Table 4:** Least squares mean (±SEM) for number of aspirated follicles and quality of recovered oocytes follicle diameters in Holstein cows receiving a diet with a 6:1-LA:ALA ratio (Low-OMG3; n = 3 pens, 25 cows, 159 oocytes) or a 2:1-LA:ALA ratio (High-OMG3; n = 3 pens, 24 cows, 148 oocytes).

Endpoint	Diet ^1^		*p*-Value		
	Low- OMG3	High- OMG3	Diet	Parity	Diet x Parity
Aspirated follicles ^2^	21.8 ± 4.2	27.1 ± 4.3	0.73	0.03	0.35
Recovered oocytes (%)	27 ± 5	21 ± 5	0.44	0.20	0.93
Grade I ^2^	2.2 ± 1.1	2.8 ± 1.1	0.44	0.36	0.35
Grade II ^2^	2.0 ± 0.7	1.9 ± 0.7	0.42	0.02	0.35
Grade III ^2^	1.25 ± 0.3	0.99 ± 0.4	0.49	0.44	0.73
Degenerated oocytes ^2^	0.5 ± 0.2	0.5 ± 0.2	0.24	0.003	0.16

^1^ Cows received a diet with a 6:1-LA:ALA ratio (**Low-OMG3**) or a 2:1-LA:ALA ratio (**High-OMG3**). Diets were isocaloric and isonitrogenous; oocyte pick up was performed at 48 days in milk. ^2^ Averaged number of structures per group.

## Data Availability

The data presented in this study are available from the corresponding author upon reasonable request.

## Supplementary Materials

The following supporting information can be downloaded at: https://www.mdpi.com/article/10.3390/ani16030395/s1, Table S1: Declared fatty acid composition in the GreatOPlus* (Naturally Better Omega-3 [NBO3], Manhattan, KS), an extruded feed product that combines flaxseed, a source of ALA, with Nannochloropsis oculata, a source of eicosapentaenoic acid (EPA); Table S2. Number of pens, cows, records, and mean lactation ± SD for the major analyses performed in the study.
