# Supplementary material for: Lower Omega-6–Omega-3 Ratio Increased Milk Production and Had Limited Effects on Early Pregnancy Development in Dairy Cattle
Source: Animals (Basel). 2026 Jan 27;16(3):395. doi: 10.3390/ani16030395 (PMC12896842; doi:10.3390/ani16030395)
Supplement: Supplementary file 1 [file animals-16-00395-s001.zip › animals-4083531-supplementary.pdf]

Supplementary Table S1. Declared fatty acid composition in the GreatOPlus* (Naturally Better Omega-3 [NBO3], Manhattan, KS), an extruded feed product that combines flaxseed, a source of ALA, with *Nannochloropsis oculata*, a source of eicosapentaenoic acid (**EPA**).

Fatty Acid (FA, Common Name)	% total fat	mg of fatty acidr per g product
Linolenic acid (ALA; 18:3 n-3)	53.32	114.64
Oleic acid (18:1 cis-9)	18.7	40.2
Linoleic acid (18:2 n-6)	15.63	33.6
Palmitic acid (16:0)	5.74	12.34
Stearic acid (18:0)	4.16	8.94
Palmitoleic acid (16:1)	2.59	5.57
Myristic acid (14:0)	0.02	0.04
EPA (20:5 n-3)	0.37	0.8
DHA (22:6 n-3)	0.11	0.24

* Biohydrogenation of alpha-linolenic acid in this product is high, with less than 5% of the amount fed reaching the small intestine [18].

Supplementary Table S2. Number of pens, cows, records, and mean lactation ± SD for the major analyses performed in the study.

	Low-OMG3				High-OMG3			
	n pen	n cows	n records	Mean ± SD lactation	n pen	n cows	n records	Mean ± SD lactation
Milk components	3	25	1050	2.2 ± 1.4	3	24	1008	2.4 ± 1.4
Milk production	3	25	3175	2.2 ± 1.5	3	24	3048	2.4 ± 1.5
Milk fatty acid profile	3	25	51	2.1 ± 1.3	3	24	47	2.4 ± 1.5
Plasma fatty acid profile	3	25	125	2.1 ± 1.3	3	24	119	2.4 ± 1.5
OPU data	3	25	159	2.8 ± 1.6	3	24	148	2.9 ± 1.6
Postpartum cyclicity	3	25	25	2.2 ± 1.4	3	24	24	2.4 ± 1.5
PAGS	3	18	126	1.9 ± 1.0	3	10	70	1.8 ± 1.3
P4	3	18	36	1.9 ± 1.0	3	10	20	1.8 ± 1.3
CL-BF%	3	18	160	1.9 ± 1.0	3	10	85	2.0 ± 1.3
CL-VOL	3	18	161	1.9 ± 1.0	3	10	86	2.0 ± 1.3
Vesicle vol	3	18	88	2.0 ± 1.0	3	10	48	2.0 ± 1.3
Embryo lenght	3	18	74	2.0 ± 1.0	3	10	42	2.0 ± 1.3
Emrbyo head	3	18	18	2.0 ± 1.0	3	10	10	1.8 ± 1.3

Pen was considered the experimental unit. Holstein cows receiving a diet with 6:1-LA:ALA ratio (Low-OMG3) or a 2:1-LA:ALA ratio (High-OMG3). Milk components (fat, protein, lactose, somatic cell count); Milk and plasma fatty acid profile (omega-6:omega-3 ratio and omega-3 content); OPU data (ovum-pick up: number and quality of recovered oocytes, lipid content and mithocondrial abundance); Postpartum cyclicity (days of first corpus luteum appearance); Pregnancy development related parameters: PAGS [pregnancy associated glycoproteins], P4 [progesterone concentrations], CLBF [corpus luteum blood flow], CLVOL [corpus luteum volume], vesicle vol [vesicle volume]). Pregnancy parameters are reported only for pregnant cows.
